# Effects of climatic factors on the net primary productivity in the source region of Yangtze River, China

**DOI:** 10.1038/s41598-020-80494-9

**Published:** 2021-01-14

**Authors:** Zhe Yuan, Yongqiang Wang, Jijun Xu, Zhiguang Wu

**Affiliations:** 1 Changjiang River Scientific Research Institute, Changjiang Water Resources Commission of the Ministry of Water Resources of China, Wuhan, China; 2 Hubei Key Laboratory of Water Resources & Eco-Environmental Sciences, Wuhan, 430010 China

**Keywords:** Ecology, Hydrology

## Abstract

The ecosystem of the Source Region of Yangtze River (SRYR) is highly susceptible to climate change. In this study, the spatial–temporal variation of NPP from 2000 to 2014 was analyzed, using outputs of Carnegie–Ames–Stanford Approach model. Then the correlation characteristics of NPP and climatic factors were evaluated. The results indicate that: (1) The average NPP in the SRYR is 100.0 gC/m^2^ from 2000 to 2014, and it shows an increasing trend from northwest to southeast. The responses of NPP to altitude varied among the regions with the altitude below 3500 m, between 3500 to 4500 m and above 4500 m, which could be attributed to the altitude associated variations of climatic factors and vegetation types; (2) The total NPP of SRYR increased by 0.18 TgC per year in the context of the warmer and wetter climate during 2000–2014. The NPP was significantly and positively correlated with annual temperature and precipitation at interannual time scales. Temperature in February, March, May and September make greater contribution to NPP than that in other months. And precipitation in July played a more crucial role in influencing NPP than that in other months; (3) Climatic factors caused the NPP to increase in most of the SRYR. Impacts of human activities were concentrated mainly in downstream region and is the primary reason for declines in NPP.

## Introduction

As an important component of global terrestrial ecosystem, vegetation plays a crucial role in in energy transfer, carbon cycle, water balance and climate regulation^1^. Its response to environment change has been considered as one of the key fields of ecological research. Net primary productivity (NPP) is defined as the net amount of carbon taken in by plants via photosynthesis, and is equal to the difference between the carbon assimilated during photosynthesis and that released during plant respiration^2,3^. It is an important indicator of ecosystem function and widely used for vegetation dynamics measure and ecological security assessment^4,5^. Remote sensing (RS) provides a convenient and efficient way for NPP estimation and promotes the NPP studies from the traditional site scale to regional or global scale^6^. Numerous ecosystem productivity models based on RS and light use efficiency (LUE) can be applied for estimating NPP, such as Carnegie–Ames–Stanford-Approach (CASA) model^7^ and Biome Biogeochemical Cycles (Biome-BGC) model^8^. The outputs of these models, namely geospatial NPP simulated results, can reveal continuous spatio-temporal patterns of vegetation. In alpine region (e.g. Tibetan Plateau), where limited availability of ground observational stations, model-based NPP estimations is an attractive alternative for vegetation changes detection.

With remote sensing applications or process-based modeling techniques, NPP trends have been explored in the worldwide. The global NPP increased by 0.19 PgC per year from 1982 to 1999^9^. And an increase of 0.03 PgC per year was found over the 15 years, between 2000 and 2014^10^. It is difficult to infer that the increasing trend has begun to slow down since 2000, because the simulation models used in the above studies were different. But the global NPP showed increase trend with some fluctuation in both periods. In China, the total NPP increased by 1.90% from the 1980s to 2015. The Huang-Huai-Hai Region has witnessed the largest increase in total NPP, followed by Loess Plateau Region and Northeast China Region^11^. The primary factors affecting NPP include climatic conditions, geochemical characteristics, ecosystem attributes and human activities. Among these factors, climatic conditions have been proven to be the dominating one^9,12^. The NPP has been significantly influenced by rising temperature and redistributed precipitation patterns in recent decades^13–15^. But primary climate factors affected the NPP differently in diverse regions. Generally, terrestrial NPP is more susceptible to temperature in middle and high latitudes while precipitation is the dominating factor in low latitudes^16^. However, some aspects of these relationships remain to be further studied and discussed, mainly due to the differences in study areas and study time periods.

The Source Region of Yangtze River (SRYR) is a typical alpine region in the western Tibetan plateau, situated at 4000 m above mean sea level. It is referred to as an important ecological security shelter zone in Three-River Headwaters region. However, The SRYR’s ecosystems and natural environment are inherently fragile and vulnerable to global warming. Similar to the whole Tibetan plateau, the SRYR has experienced significant warming trends in recent decades. The temperature increased by 0.34 °C/10a during the period from 1957 to 2013^17^ and this warming is predicted to continue in the next 30 years based on CMIP5 Climate Models^18^. It is necessary to observe the vegetation and analyze the climate change in this significant place. This research took the SRYR as the study area and explored the effects of climatic factors on NPP. In this study, the hydrothermal climatic factors refer to precipitation and temperature, which can be obtained from meteorological data and the NPP data was simulated by CASA model.

## Results

### Spatial–temporal variation of climatic factors

**Figure 1:**
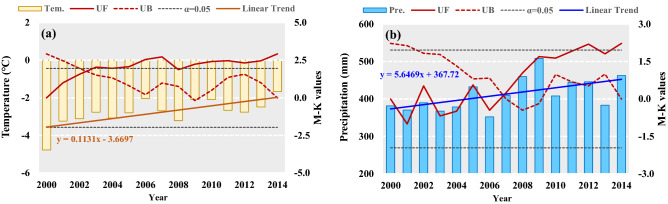
Changes in annual temperature (**a**) and precipitation (**b**) in the SRYR*. *UF and UB are test statistic values by Mann–Kendall (M–K) trend analysis. Linear Trend is obtained by linear regression method. *α* = 0.05 is the significant level.

**Figure 2:**
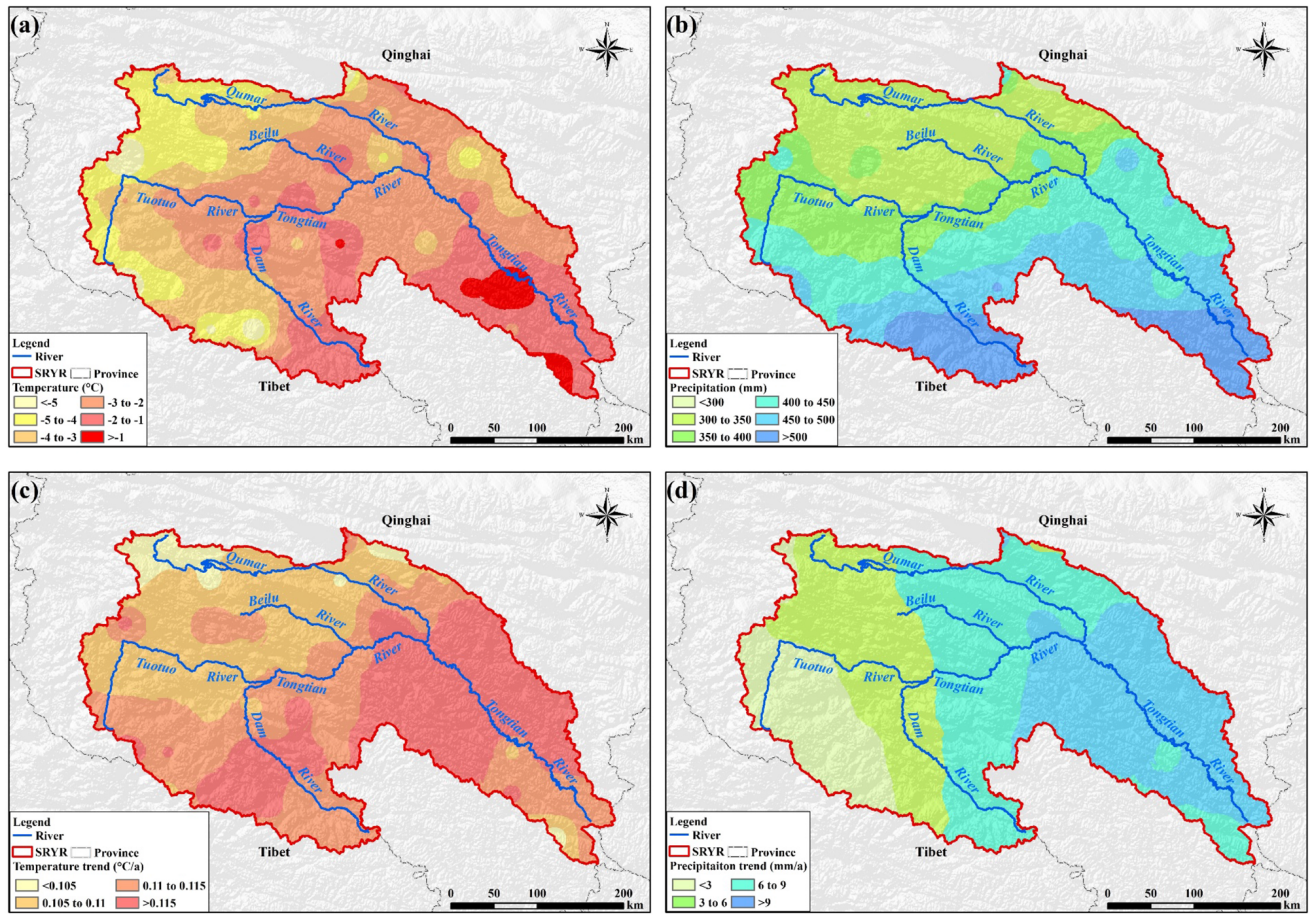
Spatial distribution of multi-year average values and change trends of climatic factors: (**a**) multi-year average temperature; (**b**) multi-year average precipitation; (**c**) Spatial trends of temperature; (**d**) Spatial trends of precipitation. Map was generated using ArcGIS 10.3 (http://www.esri.com/software/arcgis/arcgis-for-desktop).

The average annual temperature in the SRYR increased significantly at a rate of 0.113 °C per year (*p* < 0.01) on average from 2000 to 2014. A break point in 2003 can be detected With Mann–Kendall test. The multi-year average temperature was − 2.51 °C after 2003, increasing by nearly 1 °C compared with that before 2003 (Fig. 1a). An increasing trend of annual precipitation and a break point in 2007 can be found in Fig. 1b. The annual precipitation for the whole study area rose at a rate of 5.6 mm per year from 2000 to 2014 (*p* < 0.05). The average annual precipitation was 385.2 mm and 444.6 mm for period 2000 to 2007 and period 2008 to 2014, respectively, indicating a 15.4% increasing rate in recent 7 years (Fig. 1b). From the perspective of the pattern of change, the multi-year average values of temperature and precipitation were generally lower in the western SRYR and increases gradually toward the east (Fig. 2a,b). During period 2000–2014, the positive changes of temperature and precipitation can be found in all parts of the SRYR. The increase trend grew more severe in the easterly direction and a remarkable increase occurred in middle and lower reaches of the SRYR (Fig. 2c,d).

### Spatial–temporal variation of NPP

**Figure 3:**
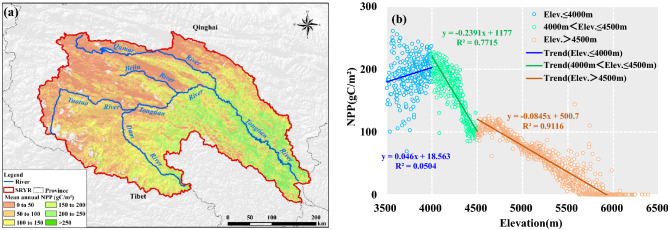
Spatial distribution of multi-year average NPP (**a**) and spatial average NPP in different elevation (**b**). Map was generated using ArcGIS 10.3 (http://www.esri.com/software/arcgis/arcgis-for-desktop).

The spatial distribution of multi-year average NPP shows that NPP is gradually lower in the northwestern SRYR and increases gradually toward the southeast, with an average value of 100.0 gC/m^2^ and a range from 0.2 to 260.9 gC/m^2^ (Fig. 3a). The spatial correlation displayed in Fig. 3b showed that there may be an exponent relation between NPP and elevation. A dispersal NPP pattern along elevation ranging from 3500 to 4000 m could be detected. When the elevation is more than 4000 and less than 4500 m, NPP is sensitive to the change of elevation. NPP reduces by 23.9 gC/m^2^ as the elevation increases by every 100 m. When the elevation is more than 4500 m, NPP is less susceptible to changes in elevation. The resulting slope indicates that a 100 m increase in elevation corresponds to a decrease in NPP by 8.4 gC/m^2^. Furthermore, the steppe is mainly located in the northwest, such as Tuotuo River Basin, Qumar River Basin and Beili River Basin, with lower NPP. However, the alpine meadow located in the southeast had higher NPP. These characteristics reflected the spatial heterogeneity of climate and terrain, and are in accordance with the gradients in humidity, temperature and elevation^19^.

**Figure 4:**
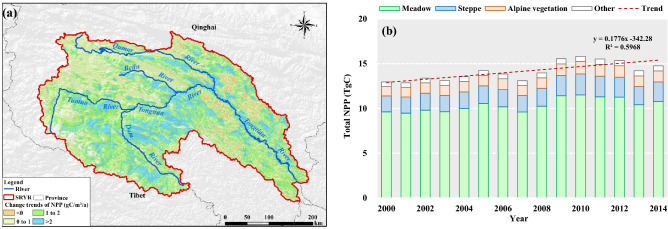
The changes in annual NPP for pixels (**a**) and the entire SRYR (**b**). Map was generated using ArcGIS 10.3 (http://www.esri.com/software/arcgis/arcgis-for-desktop).

The annual NPP in most SRYR (96.1% of total area) showed an increasing trend during the study period, with the change rate increasing from the west to the east. The most areas in the Dam River Basin and Middle stream experienced strongest positive trend (> 2.0 g C m^−2^ per year) (Fig. 4a). This result is consistent with the previous studies^11,20^. The change in annual NPP in the SRYR from 2000 to 2014 was calculated to determine the overall situation of NPP and the results are illustrated in Fig. 4b. It could be found that annual fluctuation in the total amount of NPP was not obvious during the study period, with a range of 12.85–15.81 TgC and a weak growth rate of 0.18 TgC per year. Between 2000 and 2004, the total amount of NPP remained relatively steady, and the value for the entire area was 13.2 ± 0.35 TgC. However, an obvious decreasing trend can be observed from 2004 to 2007. The value of total NPP presented in 2007 at 13.09 TgC, which is 7.4% lower than the average annual total NPP. From 2007 to 2010, total NPP in the SRYR had increased and reached the peak value of 15.81 TgC in 2010, with 11.8% higher than the multi-year average.

### Spatial variations in the impacts of climate change and human activities on NPP

**Figure 5:**
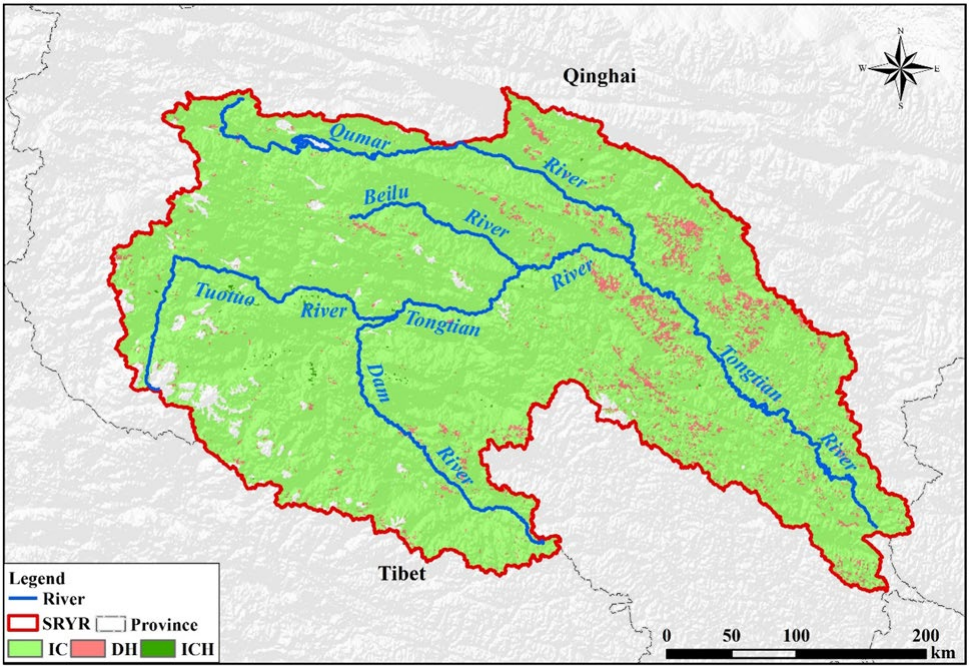
Spatial distribution of different driving forces of changes in NPP from 2000 to 2014. Map was generated using ArcGIS 10.3 (http://www.esri.com/software/arcgis/arcgis-for-desktop).

Spatial distribution of relative roles of climate change and human activities in NPP change was showed in Fig. 5. We can conclude that climate change is dominant factor impacting vegetation growth in the SRYR while the effect coming from human activities is much less. Climate change was responsible for NPP increase in 95.8% of the SRYR, whereas the human activities contributed to NPP decrease in 4.0% of the SRYR. The decreases in NPP caused by human activities were scattered, with some areas concentrated in Downstream Region. Combined impacts of climate change and human activities enhanced NPP in 0.2% of the SRYR.

### Correlations between climatic factors and NPP

**Figure 6:**
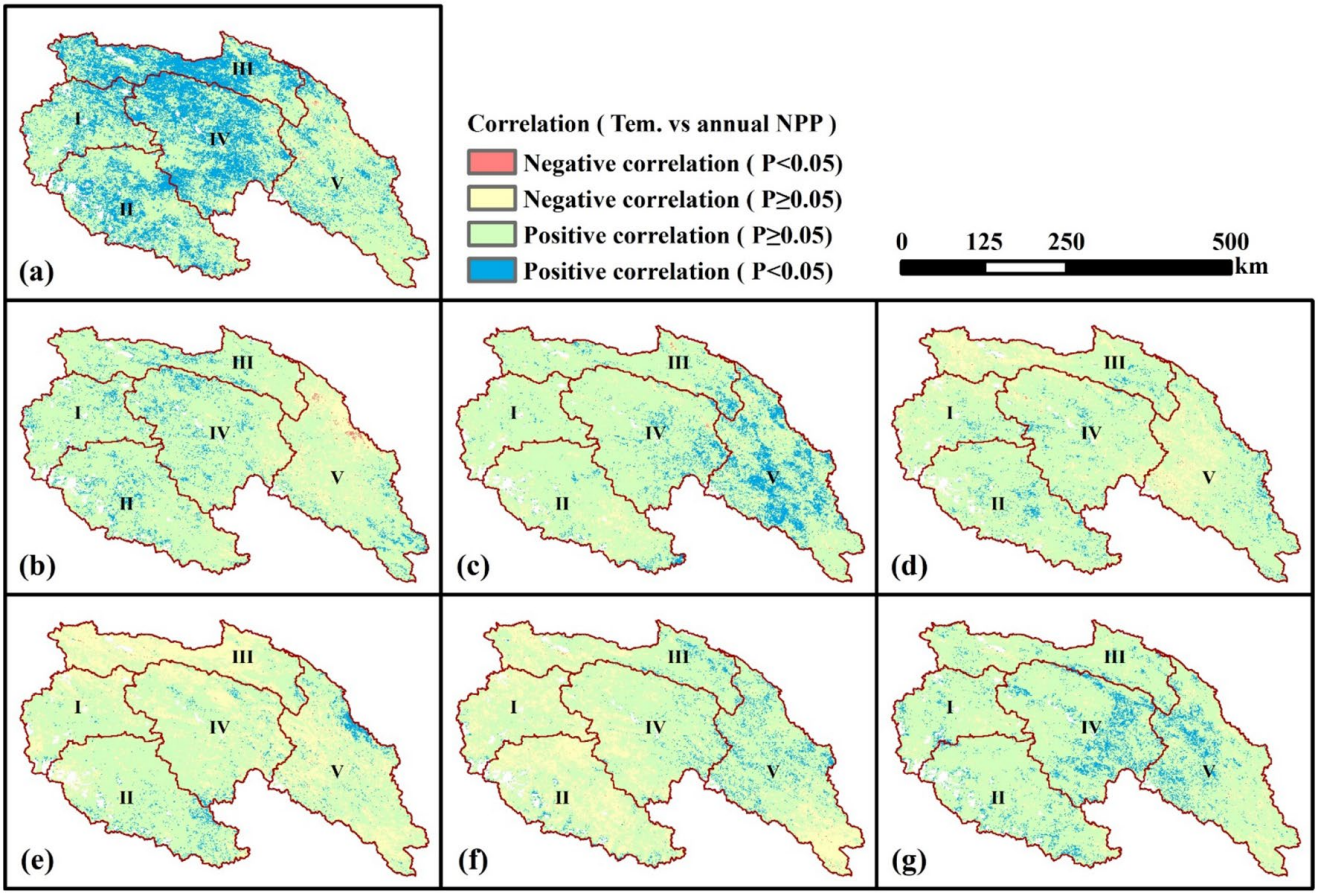
Spatial distribution of correlations between temperature and NPP. (**a**) Annual temperature and annual NPP; (**b**) April temperature and annual NPP; (**c**) May temperature and annual NPP; (**d**) June temperature and annual NPP; (**e**) July temperature and annual NPP; (**f**) August and annual NPP; (**g**) September NPP and annual NPP. Map was generated using ArcGIS 10.3 (http://www.esri.com/software/arcgis/arcgis-for-desktop).

**Figure 7:**
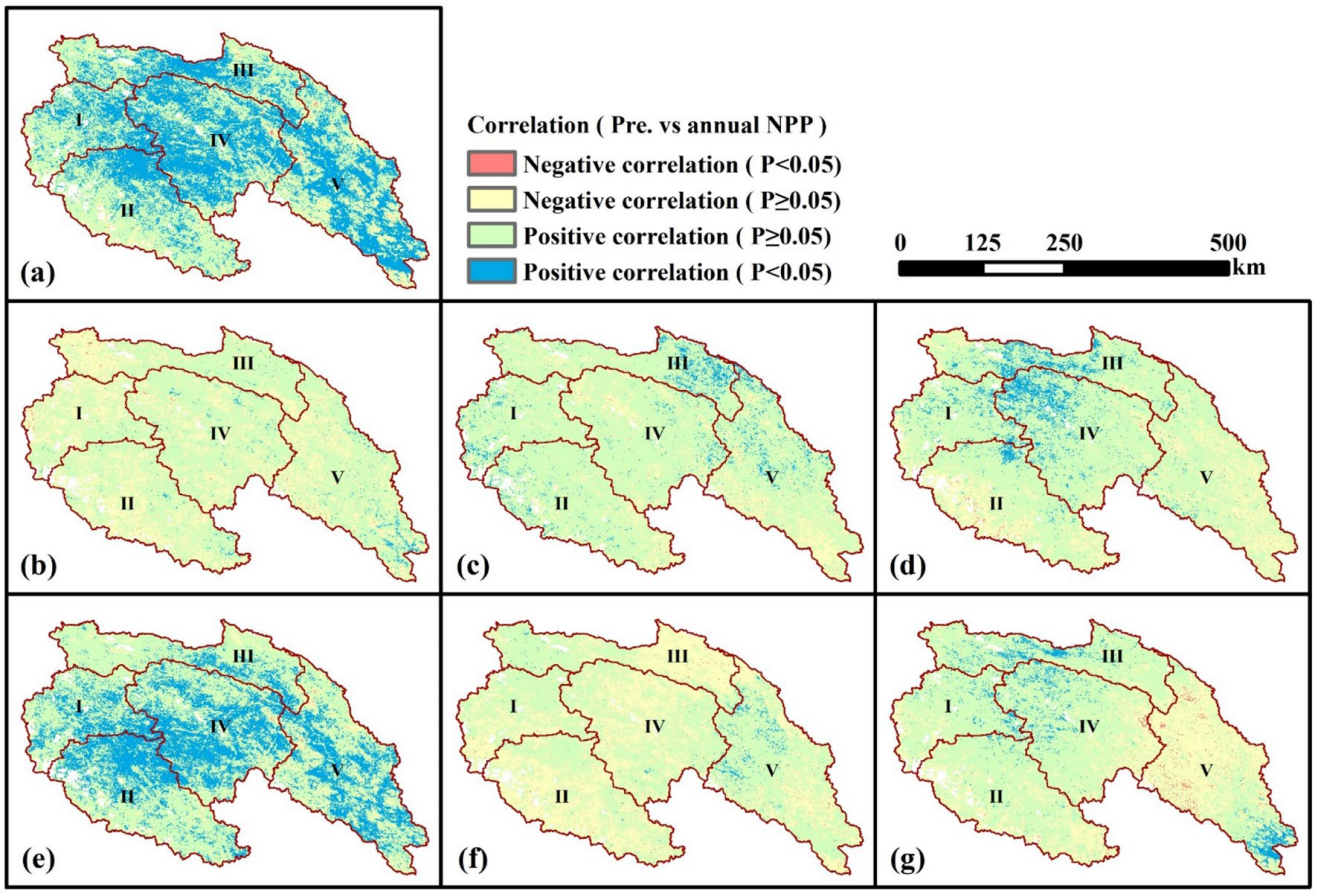
Spatial distribution of correlation coefficients between precipitation and NPP. (**a**) Annual precipitation and annual NPP; (**b**) April precipitation and annual NPP; (**c**) May precipitation and annual NPP; (**d**) June precipitation and annual NPP; (**e**) July precipitation and annual NPP; (**f**) August and annual NPP; (**g**) September NPP and annual NPP. Map was generated using ArcGIS 10.3 (http://www.esri.com/software/arcgis/arcgis-for-desktop).

The spatial distribution of correlation coefficients between climatic factors and NPP was presented by Figs. 6 and 7. It could be found that most areas of the SRYR showed a positive correlation between climatic factors and NPP. About 30.5% of the SRYR showed a significantly and positively correlation (*p* < 0.05) between annual temperature and annual NPP, primarily distributed in Qumar River Basin and Middle Stream Region (Fig. 6a). During April to September (growth season), significantly positive correlation (*p* < 0.05) between temperature and NPP was mostly observed in May (8.9%) and September (8.7%), namely the late spring and early autumn (Fig. 6c,g). Compared with temperature, the annual NPP was significantly and positively correlated with precipitation (*p* < 0.05) over more areas (40.4% of the SRYR), which were mainly found in Qumar River Basin, Middle Stream Region and Downstream Region (Fig. 7a). The significantly positive correlation between precipitation and NPP was more prevalent in July (32.1%), namely the middle summer (Fig. 7e).

### Climatic controls on NPP in different vegetation types

For the entire SRYR, annual NPP showed higher partial correlation with annual precipitation (*R* = 0.676, *p* < 0.01) than that with temperature (*R* = 0.618, *p* < 0.05). The responses of annual NPP to monthly climatic factors were varied. The annual NPP was significantly and positively correlated with temperature in September (*R* = 0.443, *p* < 0.1) and precipitation in July (*p* < 0.05). However, the partial correlations between NPP and climatic factors were insignificant in other months of growth season. For the three main vegetations, the NPP of meadow and steppe have similar responding characteristics to climate factors. By the way, the NPP of meadow and steppe has higher sensitivity to annual mean precipitation than that to annual mean temperature. Besides, annual NPP of meadow and steppe also showed the stronger partial correlation with precipitation in July during the growth season. By contrast, the annual temperature played relatively more important roles in alpine vegetation. And NPP of alpine was correlated significantly with temperature in September during the growth season (Table 1).

**Table 1:** Partial correlation coefficients between NPP and climatic factors in SRYR.

Vegetation type	Climatic factors	Period						
		Annual	Apr	May	Jun	Jul	Aug	Sep
All vegetation	Temperature	0.618**	0.354	0.415	0.302	0.169	0.219	0.443*
	Precipitation	0.676***	0.138	0.324	0.278	0.636**	− 0 .049	0.125
Meadow	Temperature	0.570**	0.334	0.425	0.329	0.214	0.218	0.451*
	Precipitation	0.686***	0.158	0.300	0.212	0.655***	− 0.052	0.079
Steppe	Temperature	0.581**	0.381	0.366	0.220	0.049	0.157	0.386
	Precipitation	0.674***	0.117	0.344	0.415	0.584**	0.018	0.303
Alpine vegetation	Temperature	0.693***	0.438	0.413	0.198	0.226	0.401	0.519**
	Precipitation	0.423	− 0.030	0.242	0.246	0.428	− 0.063	0.013

Note: *means the correlation is significant with p < 0.1; **means the correlation is significant with p < 0.05; ***means the correlation is significant with p < 0.01.

## Discussion

### Comparison of the NPP simulation and change with previous studies

The changes in vegetation in alpine regions have been investigated with NDVI, EVI or NPP. The spatial scale ranges from the source river regions to the whole Tibet Plateau. The temporal scale ranges from several years to several decades. However, researches specially on the NPP of vegetation in SRYR are relatively few. The simulated average NPP in our study was 100.0 gC/km^2^ in SRYR, which was similar to the value of 82.04 gC/km^2^ in SRYY^20^ and 143.17 gC/km^2^ in TRH^21^. Our study found an increasing rate of NPP in SRYR, indicating vegetation restoration in recent decades. Similarly, several studies detected a greening vegetation trend in Tibetan Plateau, especially in the central and eastern part^22–24^. For the change rates of the NPP, our study simulated a similar change rate (1.26 gC/km^2^ per year) with the average value (1.25 gC/km^2^ per year) on Tibetan Plateau reported by Zheng et al.^6^ Although the methods and temporal scale used in these researches were different, due to the respective characteristics of study areas, the results of this research are reliable to some degree.

### Variations in NPP along rising altitude

This research suggested that the multi-year average NPP is altitude-dependent. This characteristic is in accordance with previous studies^6,15^. When the elevation is lower than 4000 m, NPP increases as the elevation increases; when the elevation is higher than 4000 m, NPP decreases as the elevation decreases. The reason is that the area whose elevation is lower than 4000 m is mainly located in the downstream region, where the temperature will decrease as the elevation increases but the precipitation will increase as the elevation increases. What’s more, from Figs. 6 and 7, it can be seen that the impact of precipitation to NPP is bigger than that of the temperature in the downstream region. Thus, in areas where the elevation is lower than 4000 m, as the elevation increases, the changing trend of NPP is similar with that of the precipitation (increasing trend). In areas where the elevation is higher than 4000 m, temperature and precipitation will both decrease with the increase of elevation, so the NPP will decrease. In addition, for the region located in 4000–4500 m, the sensitivity of NPP to elevation is higher than that in the region located above 4500 m. Previous research also indicated that the region located in 4000–4500 m is also the transition area from seasonally frozen ground region to permafrost region^25^. This maybe the reason why NPP has different sensitivities to elevation in regions of 4000–4500 m and regions above 4500 m. Further research should be done to explore the impact mechanism.

### The effects of climatic factors on NPP

The temperature in May and September, precipitation in July were thought to have major impacts on annual NPP in SRYR compared with climatic factors in other months of growth season. Thermal conditions will greatly influence the growth of vegetation in alpine area. The temperature in spring and autumn are important elements for the beginning of vegetation growth season (BGS) and the end of vegetation growth season (EGS) respectively, which determine the length of vegetation growing season (LGS). Some researchers have found that the increase of temperature in the alpine region leaded to advanced start of BGS and delayed EGS. And the LGS prolonged as well. Accordingly, NPP of alpine vegetation also substantially increased^19,26^. This might be an important reason for the increase of NPP in SRYR. Other studies proven that summer precipitation has a greater impact on vegetation growth in alpine regions^27,28^. This is mainly because the abundant precipitation will increase soil moisture and thus improve the availability of nutrients^29^. On the contrary, if precipitation does not increase correspondingly in the context of global warming, the vegetation growth will be inhibited^30^.

**Figure 8:**
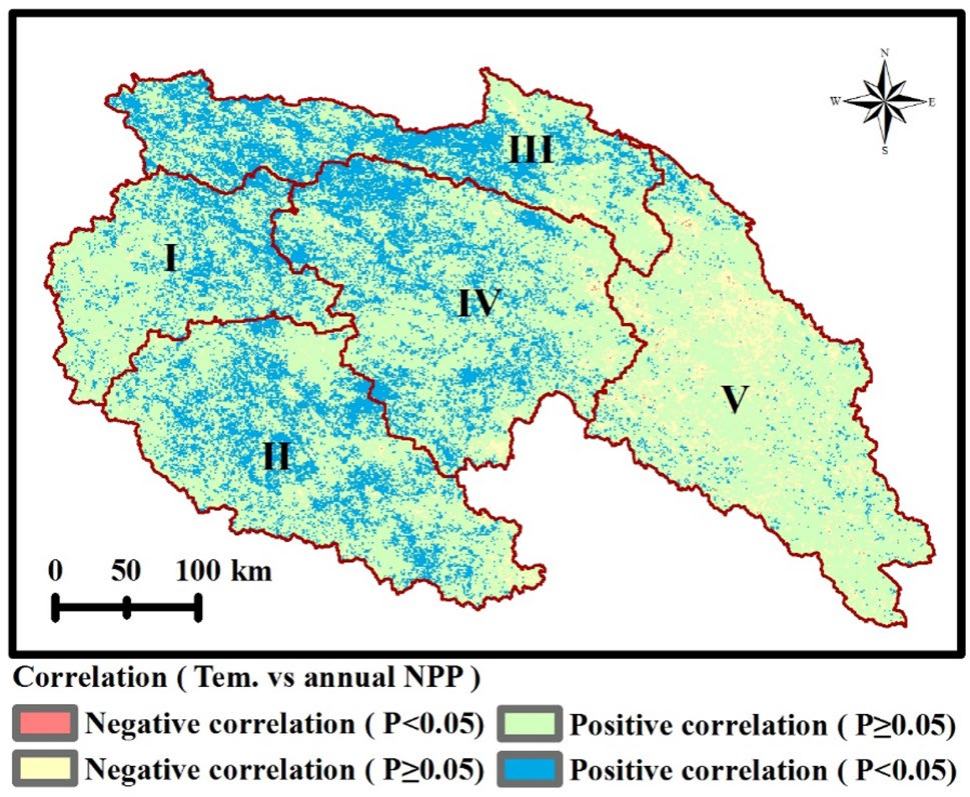
Spatial distribution of correlation coefficients between temperature in February and March and NPP. Map was generated using ArcGIS 10.3 (http://www.esri.com/software/arcgis/arcgis-for-desktop). The effects of human activities on NPP.

Table 1 showed the relationship between annual temperature and monthly temperature within the growing season between NPP. It can be seen from Table 1 that the correlation between annual NPP and annual temperature is higher than that between annual NPP and monthly temperature within the growing season. It also can be concluded that temperature which is not in the growing season can influence annual NPP. Further analysis indicates that temperature of February and March have faire correlation with annual NPP (Fig. 8). The correlation analysis shows that when the mean monthly temperature of February and March increase by 1 °C, the total NPP of the SRYR will increase by about 0.43 TgC (*R* = 0.583, *p* < 0.05). Maybe it is caused by the temperature increase in the beginning of the growing season (the end of winter and the beginning of spring), which may help to stimulate photosynthetic enzyme activities from the cold environment and ignite vegetation growth through its impacts on nutrient availability and uptake^31–33^.

**Figure 9:**
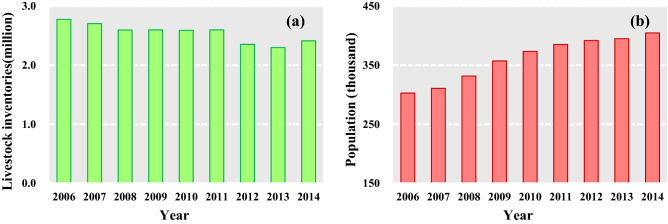
The livestock inventories (**a**) and human population (**b**) changes from 2006 to 2014 in the SRYR*. *The data was collected from Statistical Bulletin of National Economic and Social Development of Yushu Prefecture (2006–2014).

The annual NPP was highly and positively correlated with climatic variables in SRYR. We can conclude that climate change is dominant factor impacting vegetation growth in the SRYR while the effect coming from human activities is much less. The area where NPP decrease due to human activities are only 4.1% of the SRYR. Grazing is one of the primary factors to vegetation browning in this area, through vegetation cover reduction, top-layer soil degradation, soil compaction and so on^34^. In the Yushu Prefecture, mainly located in the SRYR, livestock numbers have decreased from 2.78 million in 2006 to 2.41 million in 2014 (Fig. 9a), due to grazing withdrawal policy. Although the overall grazing pressure reduced, the concentrations of grazing in specific regions (outside of the conservation area) became higher, which lead to regions of grassland degradation^22,35^. In addition, human populations have increased from 302.78 to 404.64 thousand during period 2006 to 2014 (Fig. 9b). With a rising population, the demand for material resources (e.g. milk, meat, fur products) increased continuously, which led to the livestock numbers cannot decreased significantly within a short time^36^.

## Methodology

### Study area

**Figure 10:**
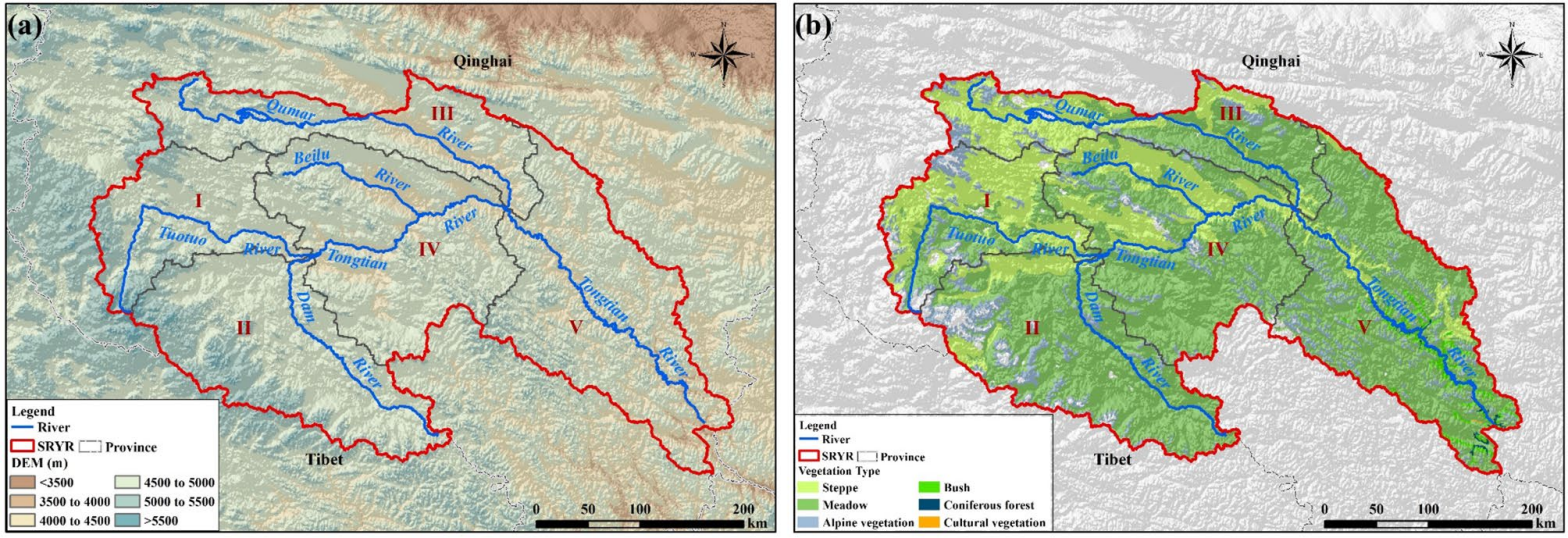
The location of Source Region of Yangtze River (**a**) and vegetation types (**b**). Map was generated using ArcGIS 10.3 (http://www.esri.com/software/arcgis/arcgis-for-desktop).

The Source Region of Yangtze River (SRYR for short, Latitude: 32° 25′ E and 35° 53′ E; Longitude: 89° 43′ E–97° 19′ E), located in the western Tibetan plateau, covers an area of 141,398 km^2^ (Fig. 10a). The elevation ranges from 6456 m in the West to 3512 m in the East, with an average of 4779 m. The SRYR belongs to transition zone from semi-arid to semi-humid alpine area. The annual temperature is − 2 to − 3 °C. Monthly mean temperature in the coldest month is − 13.0 °C and that in the warmest month is 9.7 °C. The annual temperature of the study area is 265 mm. The temperature decreases from southeast to northwest^37^. The aridity index is 3.67 in the SRYR, which means the climate is very dry. The vegetation types are mainly meadow (84,985 km^2^) and grassland (33,743 km^2^), which are 60.1% and 23.9% (Fig. 10b) of the study area respectively. We divided the SRYR into five sub-regions, including Tuotuo River Basin (I), Dam River Basin (II), Qumar River Basin (III), Middle Stream Region (IV) and Downstream Region (V).

### Datasets

The monthly NDVI data for SRYR was obtained from Resource and Environment Data Cloud Platform (RESDC, http://www.resdc.cn/). It was produced with Maximum Value Composite (MVC) approach based on the SPOT/VEGETATION NDVI data. The effects of cloud cover and non-vegetation were reduced. This dataset was at a spatial resolution of 1 km, covering the period 2000 to 2014.

The gridded meteorological data used are obtained from China Ground Precipitation 0.5° × 0.5° Grid Dataset V2.0 and China Ground Temperature 0.5° × 0.5° Grid Dataset V2.0. These datasets are provided by National Meteorological Information Center (NMIC, http://data.cma.cn/). A total of 102 grids in the SRYR and the surroundings during 2000–2014 are selected. The gridded data has been projected and resampled in order to ensure the same coordinate system and resolution with NDVI data. The NMIC also provides meteorological data of 9 meteorological stations within and around the study area, including parameters such as solar radiation, surface water, pressure, sunshine hours, wind speed and relative humidity. Grid data of the study area was interpolated by ANUSPLINE.

### NPP simulation

In this study, the NPP were simulated by CASA (Carnegie–Ames–Stanford Approach) model. The CASA model is based on the plant growing mechanism^38–40^ which can be summarized by Eq. (1).

1 $$ NPP\left( {x,t} \right) = APAR\left( {x,t} \right) \times \varepsilon \left( {x,t} \right) $$

where *x* and *t* are spatial location and time respectively, *NPP* is simulated value (gC m^−2^). *APAR* and *ε* represent absorbed photosynthetically active radiation and light use efficiency, which can be obtained by Eqs. (2) and (3).

2 $$ APAR\left( {x,t} \right) = fPAR\left( {x,t} \right) \times SOL\left( {x,t} \right) \times R $$

3 $$ \varepsilon \left( {x,t} \right) = T\left( {x,t} \right) \times W\left( {x,t} \right) \times \varepsilon_{\max } $$

where *fPAR* is the fraction of absorbed photosynthetically active radiation, *SOL* is the total solar radiation (MJ/m^2^), *R* is the fraction of solar active radiation that can be used by vegetation. T and W are temperature stress index and moisture stress factor, respectively. *ε*_max_ is maximum light utilization efficiency. Further details of the above equations can be obtained from previous studies^38–40^.

The NPP calculated by CASA model can be considered as the actual NPP which is influenced by both climate change and human activities. It can be expressed as Eq. (4).

4 $$ NPP = PNPP - HNPP $$

where *PNPP* and *HNPP* represent potential NPP and human-induced NPP, respectively. *PNPP* is only determined by climate conditions and without interference from human activities. It can be calculated by Thornthwaite Memorial model^41^, using the follows formulas:

5 $$ PNPP = 3000\left[ {1 - e^{{ - 0.0009695\left( {v - 20} \right)}} } \right] $$

6 $$ v = \frac{1.05N}{{\sqrt {1 + \left( {1.05{N \mathord{\left/ {\vphantom {N L}} \right. \kern-\nulldelimiterspace} L}} \right)^{2} } }} $$

7 $$ L = 300 + 25t + 0.05t^{3} $$

where *t*, *L*, *N* and *v* are average annual temperature (°C), annual maximum evapotranspiration (mm), annual total precipitation (mm) and average annual actual evapotranspiration (mm).

According to Eq. (4), the *HNPP* can be represented by the difference between *PNPP* and *NPP*.

### Statistical analysis

To identify the inter-annual trends of temperature (*Tem.*), precipitation (*Pre.*) and NPP, the linear regression method was adopted to eliminate the increase or decrease rate^42^, which can be calculated as follows:

8 $$ \theta_{Slope} = \frac{{n \times \sum\nolimits_{i = 1}^{n} {(i \times X_{i} ) - \sum\nolimits_{i = 1}^{n} {i\sum\nolimits_{i = 1}^{n} {X_{i} } } } }}{{n \times \sum\nolimits_{i = 1}^{n} {i^{2} - \left( {\sum\nolimits_{i = 1}^{n} i } \right)^{2} } }} $$

where *θ*_slope_ is the linear slope of the time series variable, which can be used to characterize the increase or decrease rate during a given study period; *n* is the number of years (here *n* = 15); *X*_*i*_ is the temperature, precipitation and NPP for the *i*th year (*i* = 1,2, … *n*).

A nonparametric test, Mann–Kendall (M–K) trend analysis^43,44^ was utilized to detect the break points of temperature, precipitation and NPP series in the SRYR. The test statistic *UF*_*i*_ is calculated as follows:

9 $$ \begin{array}{*{20}c} {UF_{i} = \frac{{S_{i} - E\left( {S_{i} } \right)}}{{\sqrt {Var\left( {S_{i} } \right)} }}} & {\left( {i = 1,2, \ldots ,n} \right)} \\ \end{array} $$

10 $$ \begin{array}{*{20}c} {S_{k} = \sum\limits_{i = 1}^{k} {r_{i} } } & {\left( {k = 2,3, \ldots ,n} \right)} \\ \end{array} $$

11 $$ \begin{array}{*{20}c} {ri = \left\{ {\begin{array}{*{20}c} { + 1} & {x_{i} > x_{j} } \\ 0 & {x_{i} \le x_{j} } \\ \end{array} } \right.} & {(j = 1,2, \ldots ,i - 1)} \\ \end{array} $$

where *x*_*i*_ is the variable with the sample of *n*. *E*(*S*_*k*_) and variance *Var*(*S*_*k*_) could be estimated as follows:

12 $$ E\left( {S_{i} } \right) = \frac{{i\left( {i - 1} \right)}}{4} $$

13 $$ Var\left( {S_{i} } \right) = \frac{{i\left( {i - 1} \right)\left( {2i + 5} \right)}}{72} $$

Using the same equation but in the reverse data series (*x*_*n*_, *x*_*n *− 1_, …, *x*_1_), *UF*_*i*_ could be calculated again. Defining *UB*_*i*_ = *UF*_*i*_ (*i* = *n*, *n *− 1, …, 1), we can get the curve of *UF*_*i*_ and *UB*_*i*_. If the intersection of the *UF*_*i*_ and *UB*_*i*_ curves occurs within the confidence interval, it indicates a change point^45^.

To assess the effects of temperature and precipitation on NPP in the SRYR, correlation coefficient *R* was employed to analyze the correlation between two variables (*NPP* vs. *Tem.*, *NPP* vs. *Pre.*), using the following formula:

14 $$ R_{XY} = \frac{{\sum\nolimits_{i = 1}^{n} {\left( {X_{i} - \overline{X} } \right)\left( {Y_{i} - \overline{Y} } \right)} }}{{\sqrt {\sum\nolimits_{i = 1}^{n} {\left( {X_{i} - \overline{X} } \right)^{2} \sqrt {\sum\nolimits_{i = 1}^{n} {\left( {Y_{i} - \overline{Y} } \right)^{2} } } } } }} $$

where *Y* denotes the *NPP* and *X* denotes temperature or precipitation.

The results of the statistical analysis above can be got by MATLAB.

### Identification of the relative roles of climate change and human activities in NPP

A positive *PNPP* slope indicates that vegetation growth is promoted by climate change, whereas a negative *PNPP* slope means that climate change reduced the vegetation NPP. A positive *HNPP* slope suggests that human activities have negative influence on vegetation growth and create ecological degradation, whereas a negative *HNPP* slope means that human activities contribute to vegetation growth^46^. Thus, the determinants for NPP change can be identified according to Table 2.

**Table 2:** The causes of actual NPPA change.

Method (comparing slope)			Cause of NPP change
Slope_NPPA_	Slope_PNPP_	Slope_HNPP_	
> 0	> 0	> 0	Climate change contributes to NPP increase (IC)
< 0	< 0	< 0	Climate change contributes to NPP decrease (DC)
> 0	< 0	< 0	Human activities contribute to NPP increase (IH)
< 0	> 0	> 0	Human activities contribute to NPP decrease (DH)
> 0	> 0	< 0	Climate change and human activities contribute to NPP increase (ICH)
< 0	< 0	> 0	Climate change and human activities contribute to NPP decrease (DCH)
